# Endothelial Dysfunction and Oxidative Stress in Patients with Severe Coronary Artery Disease: Does Diabetes Play a Contributing Role?

**DOI:** 10.3390/medicina61010135

**Published:** 2025-01-15

**Authors:** Alexandra Maria Boieriu, Cezar Dumitrel Luca, Carmen Daniela Neculoiu, Alina Bisoc, Diana Țînț

**Affiliations:** 1 Faculty of Medicine, Transilvania University of Brasov, 500036 Braşov, Romania; alexandra.crisan@unitbv.ro (A.M.B.);; 2 Department of Cardiology, Emergency County Hospital, 500036 Braşov, Romania; 3 “BenedekGeza” Cardiovascular Rehabilitation Hospital, 500036 Covasna, Romania; 4 Clinical Laboratory, Emergency County Hospital, 500036 Braşov, Romania; 5 Department of Cardiology, ICCO Clinics, 500036 Braşov, Romania

**Keywords:** endothelial dysfunction, oxidative stress, severe coronary artery disease, diabetes, CABG

## Abstract

*Background and Objectives*: Endothelial dysfunction (ED) and oxidative stress play major contributions in the initiation and progression of atherosclerosis. Diabetes is a pathological state associated with endothelial damage and enhanced oxidative stress. This study evaluated endothelial dysfunction and oxidative stress in patients with severe coronary artery disease (CAD) undergoing coronary artery bypass graft (CABG) surgery, comparing those with and without type 2 diabetes mellitus (T2DM). *Materials and Methods:* We included 84 patients with severe coronary artery disease (33 of whom had type 2 diabetes mellitus) who underwent clinical assessments, ultrasound, and coronaryangiography. The SYNTAXI score was calculated from the coronaryangiogram. Blood samples were collected to measure plasma serotonin (5-HT; SER) levels, as well as levels of superoxide dismutase 1(SOD-1) and lectin-like oxidized low-density lipoprotein receptor-1(LOX-1) to assess oxidative stress. Brachial flow-mediated dilation (FMD) was used as a surrogate for endothelial dysfunction (ED),along with serum concentrations of 5-HT. *Results:* The coronary atherosclerotic burden, assessed using the SYNTAX I score, was more severe in patients with CAD and associated T2DM compared to those with CAD without T2DM (30.5 (17–54) vs. 29 (17–48); *p* = 0.05). The SYNTAX score was found to be positively correlated with T2DM (*p* = 0.029; *r* = 0.238).ED measured by FMD was associated with T2DM (*p* = 0.042; *r* = −0.223), with lower FMD measurements in T2DM patients when compared with individuals without this pathology (2.43% (0.95–5.67) vs. 3.46% (1.02–6.75); *p* = 0.079). Also, in the studied population, T2DM was correlated with serum 5-HT levels (764.78 ± 201 ng/mL vs. 561.06 ± 224 ng/mL; *p* < 0.001; *r* = 0.423), with higher plasma circulating levels of 5-HT in patients with T2DM. No statistically significant differences for oxidative stress markers (SOD-1 and LOX-1) were obtained when comparing T2DM and non-T2DM patients with severe CAD. *Conclusions:* ED (as assessed by brachial FMD and serum 5-HT) is more severe in in diabetic patients with severe CAD scheduled for CABG surgery, while oxidative stress (as evaluated through serum SOD-1 and LOX-1 concentrations) was not influenced by the presence of T2DM in this specific population. The most important finding of the present study is that circulating 5-HT levels are markedly influenced by T2DM. 5-HT receptor-targeted therapy might be of interest in patients undergoing CABG, but further studies are needed to confirm this hypothesis.

## 1. Introduction

Cardiovascular disease and type 2 diabetes mellitus (T2DM) remain leading health issues associated with significant morbidity and mortality. In the quest for novel therapeutic targets, research aimed at revealing the molecular pathways involved in the initiation and progression of atherosclerosis, which is the main pathological process implicated in coronary artery disease (CAD). The link between diabetes and atherosclerosis is well established. Insulin resistance and hyperglycemia are the most important elements involved in the accelerated atherosclerosis present in T2DM [1]. Insulin resistance promotes both atherogenesis and plaque progression. The mechanisms involved are both systemic, such as dyslipidemia, hypertension, and a pro-inflammatory state, as well as the effect of disrupted insulin signaling at a cellular level (endothelial cells, vascular smooth muscle cells, and macrophages). In endothelial cells, insulin resistance inhibits nitric oxide synthetase activity and promotes leukocyte adhesion, thus promoting atherosclerosis [2]. Hyperglycemia leads to protein glycation and the formation of advanced glycation end-products (AGEs), which are substances involved in the initiation and progression of atherosclerotic lesions through multiple mechanisms, including direct cytotoxicity (oxidative stress occurring during AGE formation leads to cell damage). Receptors for AGEs (including lectin-like oxidized low-density lipoprotein receptor-1—LOX-1) are upregulated in atherosclerotic plaques in patients with T2DM. Another mechanism involves protein kinase C activation, resulting in increased vascular permeability and endothelial dysfunction (ED), mainly by decreasing nitric oxide (NO) production. Also, in patients with T2DM, increased oxidative stress is elevated due to several factors, among them glucose autoxidation, enhanced glycation, increased AGEs, the upregulation of receptors for AGEs, and the activation of the protein kinase C pathway [3].

An intact endothelial layer seems to be of the utmost importance for vascular homeostasis, as it maintains the balance in oxidative and inflammatory processes, the proliferation of vascular smooth muscle cells, vasomotion, and coagulation/fibrinolysis. Endothelial dysfunction is a pathological state that is present in cardiovascular disease, from the subclinical stages of atherosclerosis to overt chronic and acute coronary syndromes [4]. T2DM contributes to ED in both the micro- and macro-vasculature [5]. The hallmark of endothelial dysfunction is the alteration of endothelium-dependent vasodilation, primarily mediated by NO. Therefore, testing for ED mainly involves stimulating the release of NO from the endothelium. Flow-mediated dilation (FMD) is a non-invasive physical method widely used for the assessment of endothelial function. The test involves measuring the diameter of an artery using high-resolution ultrasound, before and after occluding the artery with a sphygmomanometer cuff [6].

ED is a systemic process. Hence, the evaluation of peripheral brachial FMD represents a non-invasive method used to quantify ED, and its measures correlate well with the severity of CAD evaluated based on coronary angiograms and the SYNTAX I score (Synergy Between Percutaneous Coronary Intervention with Taxus and Cardiac Surgery) [7,8].

The molecules implicated in platelet aggregation also release NO. In the absence of an endothelial barrier, the platelet-derived serotonin (or 5-hydroxyl tryptamine—5-HT) diffuses toward the underlying vascular smooth muscle, exerting important vasoconstriction. The vasoconstrictive effect of 5-HT is enhanced in atherosclerosis, which is a pathological state associated with endothelial dysfunction [9]. High 5-HT serum levels in diabetes have been associated with ED and enhanced thrombogenesis [10].

The generation of reactive oxygen species (ROS) is another key process involved in ED. One such species, cytosolic copper/zinc superoxide dismutase (SOD-1), is an antioxidant enzyme that helps mitigate oxidative damage by scavenging harmful superoxide radicals. This enzyme plays a protective role in safe guard in gendothelial cells against oxidative stress. In patients with diabetes, insulin resistance and the hyperglycemia-induced apoptosis of the vascular endothelium contribute to the development of ED [11].

The lipid peroxidation of low-density lipoprotein (LDL) plays a crucial role in the process of atherosclerosis, with oxidized LDL being a key factor. LOX-1 has been identified as the primary receptor for oxidized LDL in endothelial cells [12]. LOX-1 is upregulated in chronic inflammatory conditions, such as cardiovascular disease and T2DM [13].

This study aimed to determine whether ED is more severe in patients with type II diabetes mellitus (T2DM) and coronary artery disease (CAD) scheduled for coronary artery bypass grafting (CABG) compared to those without T2DM. The evaluation was conducted using ultrasonographic brachial flow-mediated dilation (FMD), serum serotonin (5-HT; SER) levels, and circulating oxidative stress markers, including superoxide dismutase-1 (SOD-1) and lectin-like oxidized low-density lipoprotein receptor-1 (LOX-1).

## 2. Materials and Methods

### 2.1. Design and Study Population

This prospective study included 84 adult patients with severe CAD, who were scheduled for CABG between January 2020 and June 2021. The study protocol received ethical approval from the Ethical Committee of Transylvania University (registration number 1/2.03.2019) and adhered to the principles outlined in the Helsinki Declaration and the Code for Good Clinical Practice. Informed consent was obtained in writing from all patients.

The trial included patients with severe CAD (patients with stable chronic CAD who exhibited limiting effort angina/angina equivalent that were candidates for surgical myocardial revascularization), aged >18 years, with cardiovascular risk factors (smoking, dyslipidemia, arterial hypertension, and T2DM).

Patients with acute coronary syndromes, subclavian artery stenosis, significant valvular disease, severe hepatic or renal failure, recent or active bleeding, coagulation disorders, active malignancy, carcinoid syndrome, and inflammatory diseases (including infections and autoimmune disorders) were excluded from the study. Additionally, patients diagnosed with depression and undergoing treatment with selective serotonin reuptake inhibitors and monoamine oxidase inhibitors were excluded due to the potential impact on 5-HT levels.

At admission, all patients underwent clinical, ultrasound, and coronary angiography evaluations, and blood samples were collected in accordance with the clinic’s protocol. The SYNTAX I score was determined using the number of diseased arteries, as well as the location and the aspect of atherosclerotic plaques, after performing coronary angiograms (https://syntaxscore.org/ accesed on 2 October 2021). To calculate the SYNTAX I score, a severity index is attributed to each atherosclerotic lesion identified during coronary angiography, based on stenosis location, severity, vessel tortuosity, calcification, and thrombus presence. The SYNTAX I score is a summation of these indexes; higher SYNTAX scores (>27) reflect an increased coronary atherosclerotic burden.

### 2.2. FMD Measurement

We used the brachial artery for FMD evaluation. FMD measurement was performed after six hours of fasting. Also, physical exertion, caffeine consumption, and smoking for the previous twenty-four hours were restricted [14]. Patients were kept in a quiet room in the supine posture for at least 10 min prior to the measurement. To measure FMD, we used a vascular linear probe (2D mode; 7.5–12 MHz). In order to induce reactive hyperemia, the occlusion cuff was wrapped around the forearm and inflated for five minutes to a pressure 50 mmHg higher than the systolic blood pressure. The diameter of the brachial artery was measured 3–10 cm above the antecubital fossa before the cuff was inflated, 60–90 s after maximal reactive hyperemia, and 3 min after the cuff was deflated. FMD was calculated as percentage index according to current guidelines [15]. In accordance with recent research, we considered a value <6.5% as being indicative of endothelial dysfunction [16].

### 2.3. Measurement of Serum Biomarkers

Peripheral venous blood samples were drawn after a minimum of 8 h fasting, 3 days prior to CABG. Samples were centrifuged and the supernatant was frozen at-20 degrees Celsius until the final measurements. 5-HT serum concentrations were determined by enzyme-linked immunosorbent assay (ELISA) with commercially available kits (DIAsource Immuno Assays SA, Louvain-la-Neuve, Belgium). In the first step, 5-HT is quantitatively acylated. The subsequent competitive ELISA kit uses the microtiter plate format. The antigen is bound to the solid phase of the microtiter plate. The acylated standards, controls and samples, and the solid phase-bound analyte compete for a fixed number of antiserum binding sites. After the system is in equilibrium, free antigen and free antigen–antiserum complexes are removed by washing. The antibody bound to the solid phase is detected by an anti-rabbit immunoglobulin G-peroxidase conjugate. The reaction is monitored at 450 nm. For the kits used, the measuring range for 5-HT was 10.2—2500 ng/mL, with a sensitivity of 6.2 ng/mL. The coefficient of variation (inter-assay variability) was 10.4%. LOX-1 concentrations were determined using a solid-phase sandwich ELISA method, with commercially available kits (Elabscience Biotechnology Inc., Houston, TX, USA-for LOX-1) according to the manufacturer’s specifications. The serum samples containing antigen were added to human LOX-1 antibody-coated plates. Human LOX-1 biotin conjugate was then added. Horseradish peroxidase-conjugated streptavidin was then added, binding to multiple biotin molecules for maximal signal amplification. The kits we used had a sensitivity of 2 pg/mL and the inter-assay variability was 10%. SOD-1 serum concentration was determined using commercially available kits (Elabscience Biotechnology Inc., Houston, TX, USA) following the manufacturer’s instructions. The ELISA plate provided in this kit had been pre-coated with human SOD1. During the reaction, human SOD1 in samples competed with a fixed amount of human SOD1 on the solid phase supporter for sites on the biotinylated detection antibody specific to human SOD1. Excess conjugate and unbound samples or standard were washed from the plate, and avidin conjugated to horseradish peroxidase was added to each microplate well and incubated. The enzyme-substrate reaction was terminated by the addition of stop solution and the color change was measured spectrophotometrically at a wavelength of 450 nm. The concentration of human SOD1 in the samples was then determined by comparing the optical density of the samples to the standard curve. The sensitivity was 37.5 pg/mL, with a detection range of 62.5–4000 pg/mL (0.0625–4 ng/mL). The inter-assay coefficient of variation was 10%.

All measurements were completed by the same technician who had no access to clinical information.

### 2.4. Statistical Analysis

Categorical variables were expressed as n (%); normally distributed data and skewed data of continuous variables were expressed as mean ± standard deviation (SD) and median (minimum–maximum), respectively. For skewed variables, the interquartile range was also reported. The normality of continuous variables was tested by Shapiro–Wilk’s test. To ascertain distinctions between the studied groups (T2DM versus non-T2DM), non-parametrical tests for small sample sizes were applied (Chi-square and Mann–Whitney tests). To ascertain distinctions in the analyzed data, a two-tailed Pearson correlation was applied. Statistical significance was set at *p* < 0.05. Analysis was performed using Microsoft Excel 2007 and JASP 0.19 software.

## 3. Results

Out of the 84 patients enrolled, 33 were diagnosed with T2DM. The studied population was predominantly male (26 [78.78%] with T2DM and 41 [80.39%] without T2DM), had a mean BMI of approximately 28 kg/m^2^(in both groups), and had a small percentage of smokers (9 [27.27%] in diabetics vs. 8 [15.68%] in non-diabetics). Most of the patients included had dyslipidemia (31 [93.93%] in T2DM and 49 [96.07%] in non-T2DM) and hypertension (all diabetics and 46 [90.19%] non-diabetics). Patients’ characteristics are depicted in Table 1.

**Figure 1:**
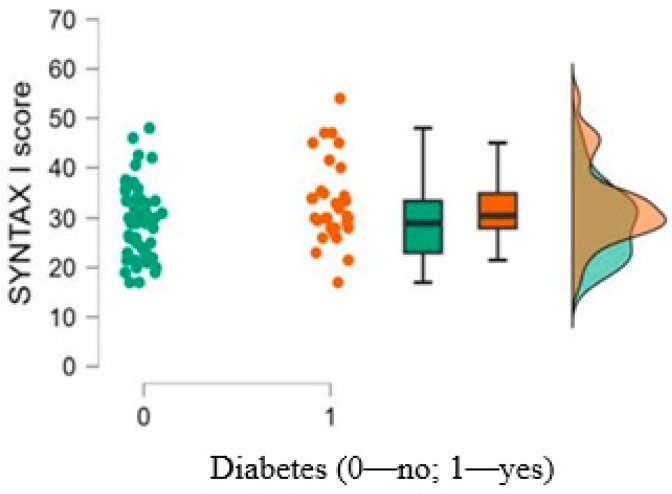
SYNTAX I score according to the presence of T2DM.

The SYNTAX I score was higher in the diabetes group (*p* = 0.050), reflecting a more severe coronary atherosclerotic burden in this category of patients (Table 1; Figure 1).

**Figure 2:**
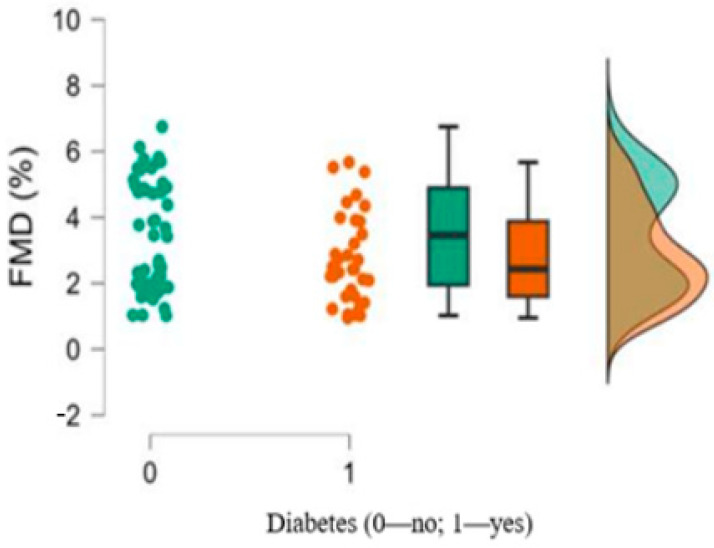
FMD measurements in patients with and without diabetes.

We observed lower brachial FMD measurements in patients with diabetes compared to those without diabetes (*p* = 0.079), though the result only approached statistical significance (Table 1; Figure 2).

**Figure 3:**
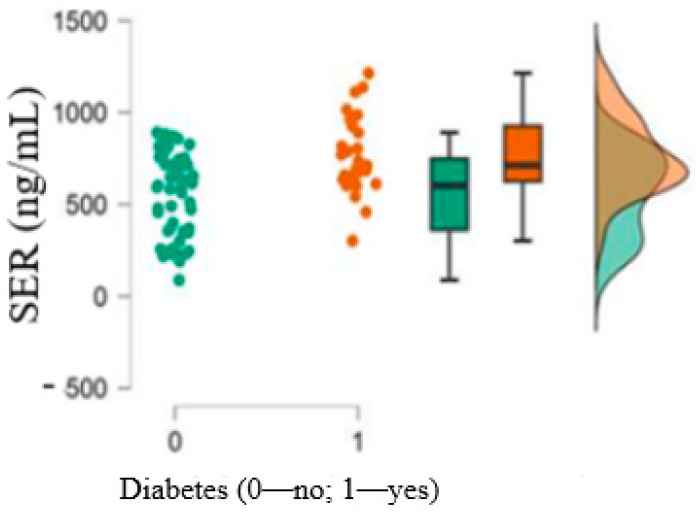
Serum serotonin (SER) levels in diabetics and non-diabetics.

The most interesting results of the present study were the higher values of serum serotonin in patients with T2DM compared to patients without T2DM (*p* < 0.001), as depicted in Figure 3.

**Figure 4:**
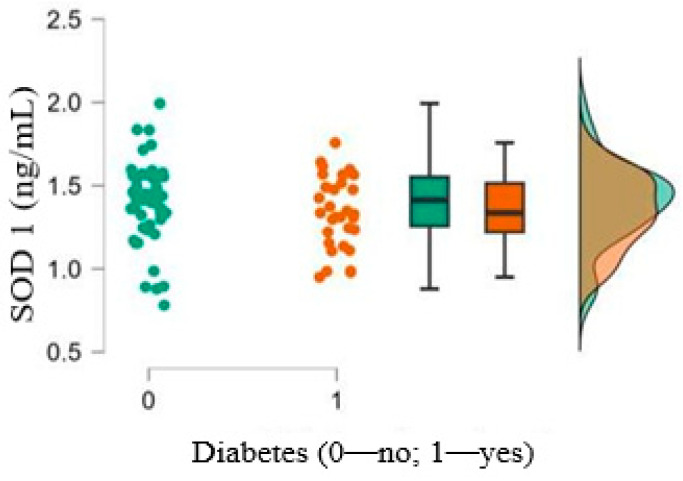
SOD1 serum levels in patients with and without T2DM.

**Figure 5:**
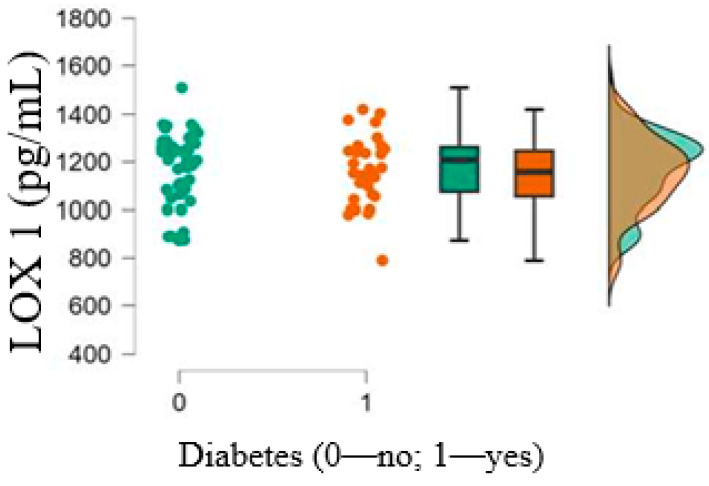
LOX1 circulating levels according to the presence of T2DM.

Oxidative stress was comparable between the groups, as the serum concentrations of SOD 1 and LOX 1 did not differ between patients with diabetes and those without diabetes (*p* = 0.362 for SOD 1 and *p* = 0.536 for LOX1). The results are shown in Figure 4 and Figure 5.

**Figure 6:**
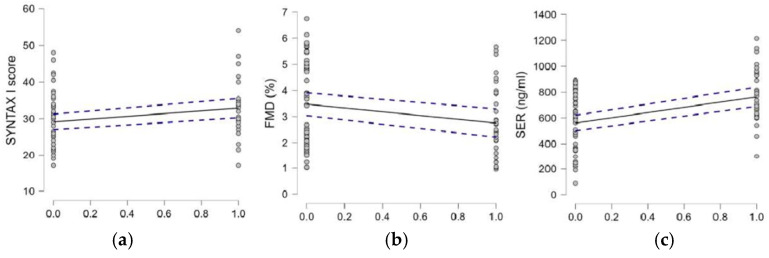
Correlations between T2DM and the studied parameters. (**a**) SYNTAX I score and diabetes (*p* = 0.029; *r* = 0.238); (**b**) FMD and diabetes (*p* = 0.042; *r* = −0.223); (**c**) FMD and serum serotonin levels (*p* < 0.001; *r* = 0.423).

Additionally, we examined whether T2DM correlated with the studied parameters (SYNTAX score; FMD; and circulating levels of 5-HT, SOD1, and LOX 1). Statistically significant correlations were found between diabetes and the SYNTAX score (Figure 6a), diabetes and FMD (Figure 6b), and diabetes and 5-HT serum concentrations (Figure 6c). A summary of these results is presented in Table 2.

## 4. Discussion

Diabetes mellitus is a prothrombotic and hypercoagulable state that is predisposed to thrombus formation. Platelets play a pivotal role in atherogenesis and its thrombotic complications [17]. Coronary atherosclerosis is more severe and diffuse in diabetic patients. Hence, the SYNTAX score, which evaluates the anatomic complexity of CAD, is higher in patients with T2DM. In the studied population, diabetes and SYNTAX score were correlated (*p* = 0.029; *r* = 0.238), with a more severe and complex atherosclerotic burden being identified in patients diagnosed with T2DM. When comparing the two groups (patients with CAD without T2DM, and patients with CAD with T2DM), the difference was also significant (*p* = 0.05).

Endothelial function is an integrated index of all atherogenic and atheroprotective factors present in an individual [18]. The endothelium is the key modulator of vascular tone and vascular homeostasis. A dysfunctional endothelium induces vasoconstriction, mainly through the reduction in NO bioavailability, and exerts pro atherogenic and pro thrombotic effects [19].

The techniques for assessing the endothelial function in the clinical setting are mainly based on the vessel’s response during reactive hyperemia, which induces endothelial-dependent relaxation by releasing NO. FMD of the peripheral vasculature is a non-invasive technique widely used in clinical practice for assessing endothelial function. Moreover, several studies have shown that it correlates well with coronary artery ED [7,8]. ED is present in all stages of CAD, promoting atherosclerosis progression and acute coronary events [20]. The present study found that ED, measured by FMD, is severe in the studied population (patients with severe CAD undergoing surgical myocardial revascularization). FMD values were below the established cutoff of 6.5% in both diabetics and non-diabetics. Hyperglycemia induces repeated alterations of intracellular metabolism and long-term changes in the structure and function of molecules through the formation of advanced glycation end products [21]. Thus, T2DM patients had a lower vasodilator response to reactive hyperemia versus patients without T2DM, even if the statistical significance was low for the analyzed population. Our study showed that FMD is influenced by T2DM (*p* = 0.042; *r* = −0.223), with reactive hyperemia being less prevalent in patients with CAD that associate diabetes; to the best of our knowledge, this research represents the first study to enroll patients with severe CAD, specifically those scheduled for CABG, and to comparatively evaluate FMD based on the presence or absence of T2DM.

ED is present from the early stages of both T2DM and CAD. However, few studies compared ED (assessed by FMD) in patients with CAD and T2DM versus patients with CAD without T2DM [22,23,24,25]. Similar to our findings, two other studies [23,24] observed that the subjects with both CAD and T2DM had lower FMD than subjects with CAD but no T2DM. The studies conducted by Bhargava et al. [22] and Simova et al. [25] observed ED across different stages of CAD. The first included Indian subjects, different from our Caucasian population. In contrast to our findings, they observed a similar degree of ED in subjects with T2DM and CAD compared with subjects who only had CAD. The latter study found no statistical difference in patients with more extensive CAD, regardless of the presence of T2DM.

Plasma serotonin levels are associated with accelerated atherosclerosis [26]. In patients with diabetes, elevated plasma 5-HT levels have been documented [27], as well as 5-HT-induced platelet aggregation [28] and the upregulation of vascular 5-HT_2A_ receptors [29]. Additionally, higher levels of 5-HT are associated with insulin resistance. In hepatocytes, gut-derived 5-HT signaling through the 5-HT2B receptor promotes gluconeogenesis. In addition, 5-HT prevents glucose uptake into hepatocytes in a glucose transporter 2-dependent manner [30]. Furthermore, 5-HT is present in the same vesicle as insulin in the beta-pancreatic cell and is associated with glycemic regulation [31]. The release of 5-HT from activated platelets is enhanced, resulting in increased plasma levels of 5-HT [32]. 5-HT, induced by a dysfunctional endothelium, promotes platelet aggregation, thrombogenesis, and angiogenesis in T2DM vascular complications through 5HT2A receptors [33]. Moreover, in the progression of atherosclerosis, 5-HT appears to act synergistically with T2DM, as it augments hyperglycemia-induced ED [10]. Subjects with CAD already have ED, hence their vascular system is easily damaged by high levels of 5-HT. Inverse correlations were previously identified between circulating 5-HT levels and ED, as was assessed through FMD and reactive hyperemia of the peripheral arterial tonometry in smokers [34]. Increased oxidative stress might therefore be a common underlying mechanism of impaired endothelial function. Furthermore, 5-HT and oxidative stress play complementary roles in the process of atherosclerosis [35].

In the population we studied, 5-HTwassignificantlycorrelatedwithT2DM (*p* < 0.001; *r* = 0.423). Also, a statistically significant difference (*p* < 0.001; Figure 3) was obtained when comparing 5-HT concentrations in patients with diabetes versus patients without diabetes. Our study is the first to evaluate the serum levels of 5-HT in patients with severe coronary artery disease (CAD) and compare the findings based on the presence of T2DM; therefore, the results may have important clinical implications.

It is now well established that selective serotonin reuptake inhibitors (SSRIs) are effective in the treatment of depression. Additionally, depression, certain personality traits [36,37], and cardiovascular disease are frequently interconnected, with inflammation and ED being common factors in both. Recent studies have therefore focused on the cardiovascular effects of SSRIs, yielding conflicting results. Some studies report protective cardiovascular effects associated with SSRIs [38,39], while others indicate worse outcomes linked to their use [40].

Sarpogrelate, which is a 5-HT2A and 5-HT 2B receptor antagonist, inhibits serotonin-induced platelet aggregation. In the search for novel therapeutic agents to further diminish the high CAD burden in T2DM, sarpogrelate has emerged as a promising molecule, reducing the total atherosclerotic plaque volume [41]. Furthermore, 5-HT has been implicated in smooth muscle cell proliferation, which is the major cause of graft failure after CABG. Several in vivo studies conducted on isolated porcine vein [42], human isolated saphenous vein [43], and human isolated internal thoracic artery [44] concluded that sarpogrelate prevents vasospasm and early graft occlusion. Further research is necessary to establish the clinical profile of patients that might benefit the most from this therapy, particularly patients with T2DM undergoing CABG surgery.

Another interesting finding of the present study was the lack of association between SOD-1 and LOX-1, as markers of oxidative stress, and diabetes. Elevated SOD-1 activity has been reported to confer protection against oxidative stress. However, even if elevated serum concentrations are present in CAD, recent studies did not report a significant correlation between plasma SOD 1 and CAD severity [45]. A possible explanation might be that elevated SOD 1 levels might be compensated for by elevated ROS formation. In diabetes, vascular injury is induced by a variety of factors, such as hyperglycemia, oxidized LDL, angiotensin II, pro-inflammatory cytokines, and altered shear stress. Previous studies suggest that the LOX-1 cellular signal transduction pathway is important for the onset and development of diabetic vasculopathy [38,46]. A possible explanation for the conflicting result in our study is that oxidative stress levels are at the highest in patients with severe CAD, irrespective of the presence of T2DM.

As previously stated, to the best of our knowledge, our study might be the first to investigate ED, through both FMD and serum 5-HT, and oxidative stress markers (LOX-1 and SOD-1) in patients scheduled for CABG. Furthermore, we highlighted the significance of the coexistence of T2DM in exacerbating ED and examined the influence of the aforementioned factors on its progression.

Several limitations of our study need mentioning. First, low statistical differences regarding the SYNTAX score and FMD measurements in the T2DM groupversusthenon-T2DM group might have been affected by the small study population. Second, even if FMD is a recognized measurement for ED, it still lacks standardization.

ED might be reversible by targeted treatment at every phase of atherosclerosis, even in the setting of established severe CAD and atherothrombotic complications.

## 5. Conclusions

This study observed that the burden of coronary artery disease (as indicated by the SYNTAX score) is more pronounced in patients with T2DM compared to those without T2DM, even within populations with severe CAD, such as individuals scheduled for CABG.

Although endothelial dysfunction (ED), assessed through FMD and circulating 5-HT levels, is more pronounced in diabetic patients compared to non-diabetic patients, the circulating levels of SOD-1 and LOX-1, which reflect oxidative stress, are not influenced by T2DM in patients with severe CAD.

Further research is needed to determine the role of 5-HT, as its levels may serve as a more reliable serum marker and therapeutic target in patients with severe CAD associated with T2DM. Measuring serum 5-HT before CABG might become useful for identifying patients in which graft vasospasm and early graft occlusion might occur postoperatively. As research is conducted on therapies targeting 5-HT, this molecule might lead to novel compounds aimed at reducing the atherosclerotic burden in CAD associated with T2DM in the general population, as well as in subjects undergoing CABG.

## Figures and Tables

**Table 1:** Patients’ characteristics according to the presence of T2DM.

Patients’ Characteristics	T2DM (*n* = 33)	Non-T2DM (*n* = 51)	*p*
1. Age (years), mean ± SD (min–max)	65.24 ± 6.96 (46–78)	65 ± 8.82 (47–88)	0.69
2. Male, n (%)	26 (78.78)	41 (80.39)	0.858
3. BMI (kg/m^2^), mean ± SD, (min–max),	28.48 ± 4.4 (20–36.3)	28.28 ± 4.17 (18.98–38.5)	0.759
4. Smoking status, n (%)	9 (27.27)	8 (15.68)	0.197
5. Dyslipidemia, n (%)	31 (93.93)	49 (96.07)	0.653
6. Hypertension, n (%)	33 (100)	46 (90.19)	*0.064*
7. SYNTAXI score, median, interquartile range (min–max)	30.5, 7.12 (17–54)	29, 10.50 (17–48)	*0.050*
8. FMD%, median, interquartile range (min–max)	2.43, 2.28 (0.95–5.67)	3.46, 2.95 (1.02–6.75)	*0.079*
9. 5-HT (ng/mL),mean ± SD (min–max)	764.78 ± 201.44 (301.75–1214.57)	561.06 ± 224.3 (87.97–891.56)	*<0.001*
10. SOD1 (ng/mL),mean ± SD (min–max)	1.34 ± 0.21 (0.95–1.75)	1.39 ± 0.24 (0.78–1.99)	0.362
11. LOX1 (pg/mL), median, interquartilerange (min–max)	1157.169, 191.90 (789.00–1420.54)	1207.10, 187.846 (874.12–1512.56)	0.536

Abbreviations—BMI: body mass index; FMD: flow-mediated dilation; SD: standard deviation; T2DM: type 2 diabetes mellitus; italics: statistical significance; 5-HT: 5-hydroxytryptamine.

**Table 2:** Correlation coefficients and statistical significance for the studied parameters.

Pearson’s Correlations							
Variable		SYNTAX Score	FMD (%)	5-HT (ng/mL)	SOD 1 (ng/mL)	LOX 1 (pg/mL)	Diabetes (0—No; 1—Yes)
1. SYNTAXI score	Pearson’s	-					
	*p*-value	-					
2. FMD (%)	Pearson’s	−0.786 ***					
	*p*-value	<0.001					
3. 5-HT	Pearson’s	0.159	−0.141	-			
(ng/mL)	*p*-value	0.149	0.200	-			
4. SOD	Pearson’s	−0.067	0.099	0.038	-		
1 (ng/mL)	*p*-value	0.547	0.372	0.731	-		
5. LOX	Pearson’s	−0.104	0.105	−0.035	0.185	-	
1 (pg/mL)	*p*-value	0.348	0.341	0.751	0.092	-	
6. Diabetes	Pearson’s	0.238 *	−0.223 *	0.423 ***	−0.106	−0.048	-
(0—no; 1—yes)	*p*-value	0.029	0.042	<0.001	0.338	0.666	-

* *p* < 0.05; *** *p*< 0.001.

## Data Availability

The datasheets are part of an ongoing study; requests for datasets should be directed to alexandra.crisan@unitbv.ro.
